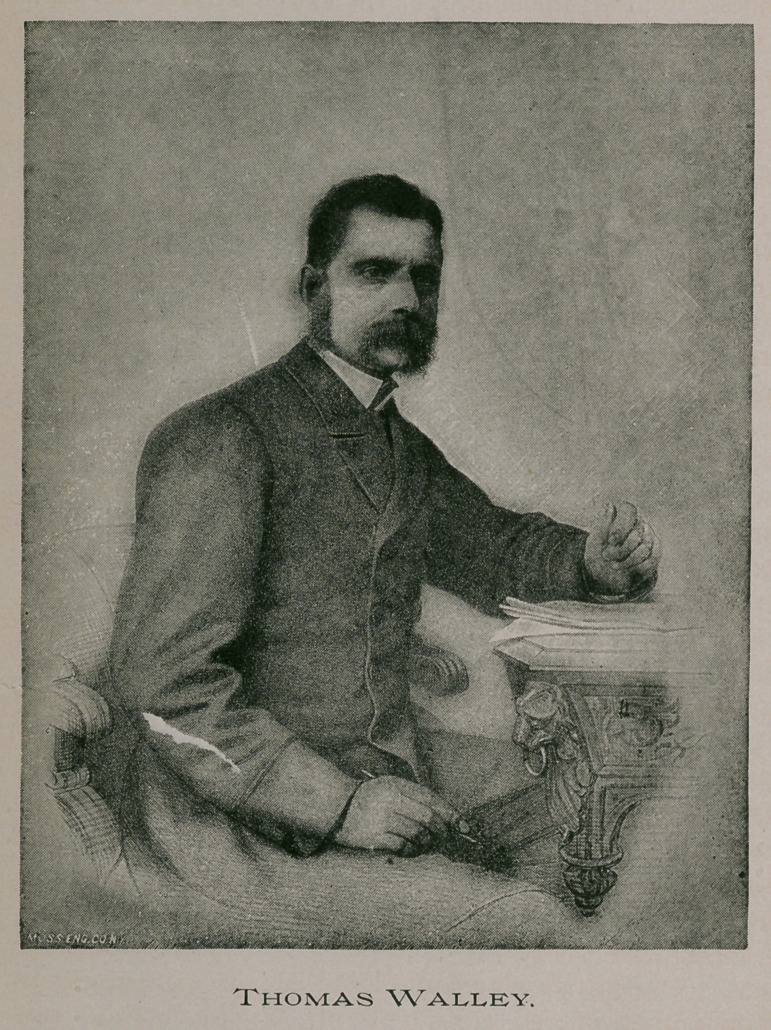# Thomas Walley, M. R. C. V. S.

**Published:** 1886-10

**Authors:** 


THOMAS WALLEY.

### Art. XXI.—THOMAS WALLEY, M. R. C. V. S.

PRINCIPAL ROYAL (DICK), VETERINARY COLLEGE, EDINBURGH.

Professor Walley, the subject of the accompanying portrait, first saw the light at Old Springs, Market Drayton, North Shropshire, England. His progenitors for several generations were distinguished agriculturists in his native country. It may be mentioned in passing that the Professor's maternal grandmother was cousin of the beautiful Sarah Hoggins, whose romantic history Tennyson has celebrated in his well-known poem "the Lord of Burleigh."

Except that his very early years were spent at the two best local schools the Professor might almost be said, as regards general attainments, to be self-educated. While still very young, he was sent, contrary to his own desire, to the establishment of a well-known merchant in Chester; but three years afterward, when at the death of his father he was allowed to follow the bent of his own inclination, he abandoned this occupation, and, having given to him the choice of two professions—medical or veterinary—he chose the latter, and was forthwith apprenticed to Mr. Bampfield Kettle, F. R. C. V. S., who was then, as now, conducting an extensive mixed practice in the County of Shropshire. Mr. Walley remained under the tuition of Mr. Kettle for more than three years, and during this period he may be said to have been thoroughly trained as a veterinary surgeon, and to have had those habits of industry, perseverance, and painstaking study, which have mainly contributed to raise him to his present prominent position in the profession. Another year was spent as assistant in the practice of Mr. Stanley, of Birmingham, and in October, 1861, he entered the Royal Veterinary College, London, as a student. During his curriculum, out of the comparatively few honors then offered to students, Mr. Walley obtained two silver medals and a certificate of honor. One of these medals was for a dissection that is still preserved in the college museum. During the latter part of his curriculum Mr. Walley acted as prosector to the late Professor Varnell, for whom he entertained a great affection.

In April, 1863, Mr. Walley obtained the diploma of the Royal College of Veterinary Surgeons, and for eight years subsequently he practiced his profession at Welshpool, West Derby, and Manchester. In 1871, Mr. Walley was appointed, after a competitive examination, to the chair of Cattle Pathology, in the Royal (Dick's) Veterinary College, Edinburgh. About a year later he was called upon to teach the additional subject of anatomy, and in 1874 he was elected to the vacant principalship of the college, which office he continues to hold at the present time. Since Professor Walley was appointed to the principalship the history of the college has been one of unbroken prosperity. The number of students has more than trebled, and at the present time, the accommodation having become quite inadequate, the college is being rebuilt on a scale that will make it the most complete and efficient for teaching purposes in Great Britain. A very large share of the labor of teaching is discharged by the Professor, who now personally conducts separate courses of tuition on Equine Pathology, Cattle Pathology, and Materia Medica Therapeutics. He occupies the honorary position of professor of Cattle Pathology to the Highland and Agricultural society, and holds appointments as veterinary inspector to the Privy Council and to the Lord Provost and Magistrates of the city of Edinburgh.

Professor Walley has been intimately and actively associated with all the recent movements having for their object the advancement of the profession. He was a Vice-President of the Council of the R. C. V. S., from 1876–79, and has been a member of the Council since 1880. During the year 1884–85, he held the office of President of the R. C. V. S., and for the current year he is President of the National Veterinary Association. He is also an honorary fellow of several British and of the Montreal V. M. Association. Professor Walley is the author of "The Veterinarian's Pocket Conspectus" (now out of print) and of "The Four Bovine Scourges," besides which he has written original articles too numerous to mention, the majority of which have been read before the various Veterinary Medical Associations, or printed in the columns of the professional journals. But the Professor's position in the veterinary profession is not to be measured by the amount of his contributions to current veterinary literature. In his capacity as Director of the Dick Veterinary College he has done excellent work in extending and perfecting the veterinary curriculum. As an instance of his influence for good in this direction we may state that he was the first to recognize the extreme importance of a practical knowledge of pathological histology to the modern veterinary student, and he was the first veterinary teacher in Great Britain to institute a regular class for the practical teaching of that most essential subject. And the effect of this advance in teaching was not confined to his own school, for that subject is now, we believe, taught with more or less thoroughness in all the other British veterinary schools. Notwithstanding the exacting nature of Professor Walley's daily duties, he finds time to read most extensively. His constant effort is to keep himself fully abreast of all improvements and discoveries in medical and veterinary science, and knowledge thus acquired is at once infused into his own teaching.

But there is another respect in which Professor Walley's position as a teacher is exercising a powerful influence for good to the veterinary profession in Great Britain. We have more than once heard the Professor's character summed up by a professional brother in the statement "Professor Walley is a gentleman," the epithet being applied in the simplest and best sense. In his intercourse with his clients, with his pupils, and with his professional brethren, Professor Walley is one who would scorn to do an action that would not bear the light of day. To his students, and to all who come into contact with him, he inculcates by precept and by example a rigid and honorable professional etiquette. Within the college walls he is a strict, but not a stern disciplinarian, and it is literally true that he is beloved by his students. Professor Walley has attained his already pre-eminent position while he is yet a comparatively young man. He is already, especially with the younger section of the profession, one of the most popular veterinarians in his native country. We sincerely trust that he may long be spared to continue the work he is doing with much honor to himself and great benefit to his profession.

## Figures and Tables

**Figure f1:**